# British Medicinal Plants

**Published:** 1891-04

**Authors:** Alfred Heath

**Affiliations:** London, England


### BRITISH MEDICINAL PLANTS.

ALFRED HEATH, M. D., F. L. S., LONDON, ENGLAND.

### ORDER 15.—MALVACEÆ.

*Malva moschata* (Musk Mallow).—The most elegant of our native mallows, flowering very freely, and throwing out a faint perfume of musk toward evening. It was at one time famous as a remedy for the gravel and for suppression of urine, also in some forms of headache. The seed is the part generally used. There is no proving of the drug, but if proved it might perhaps be a valuable medicine.

*Malva sylvestris* (Common Mallow).—There is also no "proving" of this plant. It is used in the same way as the species previously mentioned, to promote the discharge of urine and to relieve stranguary, gravel, etc. It is also similar in its effects to the following:

*Althœa officinalis* (Marsh Mallow).—Found in marshy places, particularly near the sea. The roots contain a large amount of mucilaginous matter which is extracted by boiling in water. The virtues of this plant are beyond question, and it ought to be carefully and completely "proved." It has been found useful in offensive diarrhœa with violent pains in the bowels. It is largely used in consumptive coughs, pleurisy, and other chest and lung diseases. It helps women in labor, and increases the secretion of milk in the breasts. Pliny says: "that whoever shall take a spoonful of the Mallows shall that day be free from all diseases *that may come unto him.*" It is useful against the stings of bees, wasps, etc., the bruised leaf only being applied; also for inflammations of various kinds it is very cooling. For roughness of the skin, drandruff, falling off of the hair, etc., this Mallow is often very useful.

### Order 16.—Tilaceæ.

*Tilia Europœa* * (common name, Lime or Linden Tree).—Every one knows the lime tree, of which we have three kinds common to this country—namely, *T. intermedia*, *T. parvifolia*, and *T. grandifolia*, all supposed by botanists to be varieties of *T. Europœa*, of which there are also several other varieties. Of the three varieties mentioned, *T. parvifolia* is undoubtedly indigenous; respecting the others there is some doubt. The inner bark, called bast, is made into mats; the Russian peasants make shoes, ropes, etc., of it. Jute, an exceedingly valuable fibre, is made from another member of this order (*corchorus capsularis*). In medicine we use the flowers of the Lime, whose delicious

---

* I take this opportunity of saying that provers of and introducers of new drugs are often not sufficiently explicit in describing plants of which there are several *varieties*, and if they do not clearly state which of the varieties they used, the pharmacist cannot be blamed if he uses either of them, or the one most easily obtained. Some may answer that it makes very little difference, but I contend that it *may* make all the difference, and to be exact in prescribing according to the "law of similars," it is necessary to be exact in describing, as well as preparing, the remedy. With respect to *Tilia Europœa* we are not told which variety is to be used, and, for aught we know, in the absence of separate provings, they may all produce some different symptoms.

perfume loads the air in June and July, attracting countless numbers of bees to the honey-like nectar contained in them. The *Tilia* was formerly used in various kinds of headaches, affections of the nerves, apoplexy, epilepsy, vertigo, palpitation, of the heart, etc. There is a proving in Allen's *Materia Medica.* Some of the symptoms produced are "heat in the head, vertigo, with tottering on turning the head, accompanied with obscuration of sight."

### ORDER 17.—HYPERICACEÆ.

*Hypericum Perforatum* (St. John's Wort).—Found on roadsides and hedge-banks, etc., in July and August. This plant was formerly held in great esteem, and was used internally in a great variety of diseases, and externally as an anodyne, and to resolve tumors. At one time it was supposed, and not without reason, that madmen were possessed of the devil, and this plant was found so successful in curing that disorder that it had the title of *Fuga Dœmonum,* as curing dæmoniacs. It was also given in hysteria, and as a remedy for burns and stings of insects.

There is a good proving of this drug. It *produces,* when taken internally, a great variety of symptoms, of which the following are some of the most important: "Mistakes in writing, omission of letters; forgetfulness of what it is desired to say; confusion; increase of intellectual power; excitement of brain; sees spectres; delirium; singing followed by weeping, and loud screaming; anxiety, melancholy, irritability. It removes consequences of fright; effects of shock, it causes vertigo at night; headache in the morning, with tearing stitches in the brain; a sensation as if the head were elongated; is useful in fractured skull, bone splinters, etc.; causes sties on lower left eye-lid; severe aching in decayed teeth at night, relieved by lying on the painful part; a desire for warm drinks, wine, pickles; absence of taste; many symptoms of disturbance of stomach and bowels; attack of spasmodic asthma with changes of the weather from clear to damp, or before storms, especially indicated upon lesion of the spinal cord by a fall years before; useful in meningitis.

Morning dry cough with prostration; whooping cough worse from six to ten P. M. Nervous system much affected after a fall. The slightest motion of the arms or neck extorts cries; cervical vertebræ very sensitive to touch; entire spine tender, bad consequences of spinal concussion; violent pains and inability to walk or stoop after a fall on bottom of back (coccyx). Various rheumatic pains in upper and lower limbs, with weakness and trembling in all the limbs; great dread of motion. The "prover" would not walk, screamed when lifted. It causes great nervous depression following wounds; next to the nervous tissues the joints are affected; all the joints feel bruised.

It is useful to prevent lockjaw from wounds in soles of feet, fingers, or palms of hands; convulsions from blows on the head, or after every slight hurt; epileptic spasms after injury; injuries to parts rich in sentient nerves, especially fingers, toes, nails, and lacerations, where the intolerable pain shows that nerves are severely involved. Useful in punctured wounds, which feel very sore, rat-bites, etc.; from crushed fingers, especially the tips, lacerated nerves with excruciating pains, painful wounds before suppuration, very painful bunions and corns.

*Hypericum Perforatum* is one of, if not the most important remedy for injury to nerves. It is one of the commonest plants, but many of the hypericums are much like it in general appearance, and great care should be exercised in collecting it, and the tincture should *always* be made from the fresh flowering plant.

*Hypericum Androsœnum* (Tutsan).—Found in thickets, etc. This plant is not so common as the preceding, and although very well known in the country, its virtues are not so well known. Its marvelous power as a wound-wort entitles it to rank as high as any of the order, and it *ought* to be "proved." The flowers are of a beautiful golden yellow, and when rubbed stain the hands red. The whole plant in the autumn becomes a blood-red color, and looks very beautiful. The leaves are wonderful in curing fresh wounds, scarcely anything equals them. The young leaves at the top are best; when bound on the wound they stop the bleeding, and very speedily cure. Many plants are famed as wound-worts but the effect of Tutsan is surprising. There is no proving.

### Order 18.—Aceraceæ.

*Acer Pseudo Platanus* (the Sycamore).—This tree is very common in England. Every school-boy knows the sycamore. The bark of the young branches is so easily moved on account of the quantity of sap that it is in general use amongst boys for making whistles. It has not been much used in medicine. The juice is anti-scorbutic. It has been used in obstruction of the liver and spleen, and to ease pain in connection with such disturbances. Like all the maples, the juice yields, on drying, a large amount of saccharine matter or molasses, similar to the sugar-maple of New England and Canada, but not to such an extent. There is no proving.

### Order 19.—Geraniaceæ.

*Geranium Robertianum* (Herb Robert, Stinking Crane's-bill).—A pretty little plant with pink flowers and stem, and sometimes red leaves, but having a very rank, unpleasant smell. It was formerly held in great esteem as an external application in erysipelatous inflammation, mastodynia, or pain in the breast. The plant is very astringent, and is given to cattle when they make bloody water, or have the bloody flux. It is an excellent wound herb used externally or internally. An ointment made from the leaves is good for sore breasts, and has been found serviceable in the treatment of scrofulous and cancerous swellings. It has been found to give relief in stone and gravel. Cattle have been reported as being cured of what farmers call "black-water" and of the bloody flux by a preparation of this plant, after all other remedies had failed. It stops overflow of the menses, bloody stools, and all other hemorrhages. In this plant and the Tutsan we have another instance of what I have before referred to as the "Doctrine of signatures." At certain seasons of the year they both turn of a *blood-red color*, and it is remarkable that they are the two best remedies the fields produce for outward and inward bleedings. There is no proving.

